# Transverse versus vertical skin incision for planned cesarean hysterectomy: does it matter?

**DOI:** 10.1186/s12884-020-2768-7

**Published:** 2020-01-31

**Authors:** Alec Szlachta-McGinn, Jenny Mei, Khalil Tabsh, Yalda Afshar

**Affiliations:** 1 Department of Obstetrics and Gynecology, University of California, Los Angeles, CA USA; 2 Division of Maternal Fetal Medicine, Department of Obstetrics and Gynecology, University of California, 10833 Le Conte Avenue, Room 27-139 CHS, Los Angeles, CA 90095-1740 USA

## Abstract

**Background:**

To investigate differences in perioperative outcomes by type of skin incision, transverse versus vertical, for planned cesarean hysterectomy for placenta accreta spectrum (PAS).

**Methods:**

A retrospective cohort study of all women who underwent a planned cesarean hysterectomy for abnormal placentation at a single academic medical center over 5 years. The Student’s t-test was used for continuous variables and Fisher’s exact test compared categorical variables. Continuous data were presented as median and compared using the Wilcoxon-rank sum test.

**Results:**

Forty-two planned cesarean hysterectomies were identified. A transverse skin incision was made in 43% (*n* = 18); a vertical skin incision was made in 57% (*n* = 24). Skin incision was independent of BMI (30.3 vs 30.8 kg/m^2^, *p* = 0.37), placental location (*p* = 0.82), and PAS-subtype (*p* = 0.26). Mean estimated blood loss (EBL) was 2.73 l (L) (range 0.5–20) and was not significantly different between transverse and vertical skin incision (2.6 L vs 2.8 L, *p* = 0.8). There was significantly shorter operative time with transverse skin incision (180 vs 238 min, *p* = 0.03), with no difference in intraoperative complications, including cystotomy (*p* = 0.22) and ureteral injury (*p* = 0.73). Postoperatively, there was no difference in maternal length of stay (4.8 vs 4.4 days, *p* = 0.74) or post-operative opioid use (117 vs 180 morphine equivalents, *p* = 0.31).

**Conclusion:**

Transverse skin incision is associated with shorter operative time for patients undergoing planned cesarean hysterectomy. There was no difference in EBL, intraoperative complications, postoperative length of stay, or opioid use. Given an increasing rate of cesarean hysterectomy, we should consider variables that optimize maternal outcomes and resource utilization.

## Background

Placenta accreta spectrum (PAS), formerly known as morbidly adherent placentation, is an increasingly common obstetrical complication associated with maternal hemorrhage and subsequent morbidity and mortality worldwide [1, 2]. While the rate of maternal mortality continues to rise, hemorrhage remains a leading cause [1–5]. PAS is classified on the invasiveness of trophoblastic tissue past the maternal decidua into the uterine myometrium; namely, placenta accreta, increta, and percreta [1, 6, 7]. Both a history of previous cesarean delivery (CD) and placenta previa are risk factors for PAS [8]. Studies of the incidence of PAS in the United States range from 1 in 272 pregnancies [9] to 1 in 731 pregnancies [10], and this risk increases with concurrent placenta previa and increasing number of prior cesarean deliveries [6, 9–12]. As the number of CDs increases in the United States, the number of pregnancies affected by PAS has increased [13].

The antepartum diagnosis of PAS is critical to optimizing delivery planning given the significant maternal and neonatal risks. Currently, the most widely accepted delivery approach to PAS is planned cesarean hysterectomy at 34 0/7 to 35 6/7 with the placenta left in situ after delivery of the fetus [13–15]. Complications associated with PAS include life-threatening hemorrhage necessitating urgent cesarean hysterectomy, damage to surrounding organs including the bowel, ureters, and bladder at time of hysterectomy, severe coagulopathy, transfusion-related complications, intensive care unit admission, and maternal death [2, 13]. Multidisciplinary delivery planning teams are recommended to improve maternal and neonatal morbidity [2, 13, 16].

There are currently no studies comparing maternal and fetal outcomes related to type of skin, midline vertical versus Pfannenstiel skin incision, for planned cesarean hysterectomy with prenatally suspected PAS. Rather, skin incision is left to surgeon preference [2, 13, 16]. The aim of this study is to compare perioperative and postoperative outcomes of planned cesarean hysterectomies performed by midline vertical versus Pfanenstiel skin incision to better guide clinical practice in optimizing maternal and neonatal outcomes in pregnancies complicated by PAS.

## Methods

A retrospective cohort study was performed utilizing all birth records to include all births for planned cesarean hysterectomy secondary to PAS at a single tertiary care academic medical center over 5 years (January 1, 2014 - July 30, 2018. Institutional review board (IRB) approval was obtained from the University of California, Los Angeles (IRB #18–000872). Planned cesarean hysterectomy was defined as antepartum suspected placenta accreta spectrum whereby a delivery plan had been established involving a multidisciplinary team, including obstetrics, perinatology, interventional radiology, urology, and anesthesia. Conservative management of PAS was excluded. Patients with undiagnosed PAS at time of delivery or patients who underwent emergent cesarean hysterectomy due to heavy vaginal bleeding with concurrent maternal hemodynamic instability and/or category II or III fetal heart tracing were excluded from analysis.

Antepartum diagnosis of PAS was made according to established sonographic features by board-certified maternal fetal medicine physicians at a single academic center. Sonographic features included loss of normal retroplacental hypoechoic zone; multiple vascular lacunae within the placenta; blood vessels or placental tissue bridging uterine-placental margin, myometrial-bladder interface, or crossing uterine serosa; retroplacental myometrial thickness of < 1 mm; and/or numerous coherent vessels visualized with 3-dimensional power Doppler in basal view [8, 13].

Forty-two patients met inclusion criteria for planned cesarean hysterectomy for placenta accreta spectrum. Records were reviewed, and demographic and outcome data were chart abstracted from antepartum notes, pre-operative imaging results, operative reports, and postpartum notes. Demographic data included maternal age, race, ethnicity, body mass index (BMI), insurance type, gravidity, and parity. Pertinent medical and surgical history data included number of prior cesarean deliveries and prior uterine surgeries. Preoperative diagnosis of PAS, presence of placenta previa, and placenta location was obtained by review of preoperative imaging. Operative reports were reviewed for gestational age at delivery, type of skin incision, type of anesthesia, estimated blood loss (defined in liters (L)), blood products transfusion requirements, surgery length (defined in minutes as time from skin incision to skin closure), and intra-operative complications. Post-operative records were reviewed for length of intensive care unit (ICU) stay (measured in days), length of hospital stay (measured in days), opioid use (in terms of morphine equivalents), wound complications, and maternal death.

The Student’s t-test was used to analyze continuous variables and Fisher’s exact test compared categorical variables. Continuous data were presented as median (interquartile range [IQR]) and compared using the Wilcoxon-rank sum test. For all analyses, *p*-values were two-way and the level of statistical significance was set at *p* < 0.05.

## Results

Forty-two planned cesarean hysterectomies for PAS were identified. Demographic data are presented in Table 1. The median maternal age at delivery was 33 years (IQR 30–36.8), and the median maternal body mass index was 33.0 kg/m^2^ (IQR 26.0–35.5). The majority of women were Caucasian (85.7%), 2.4% Black, and 11.9% identified as other. Fifty percent of patients were of Hispanic ethnicity, 40.5% were non-Hispanic White, 2.4% identified as other, and 7.1% were unknown. Forty-five percent (*n* = 19) of patients had an antenatal diagnosis of placenta accreta based on imaging, while 19% (*n* = 8) had an antenatal diagnosis of placenta increta, and 29% (*n* = 12) had an antenatal diagnosis of placenta percreta. The remaining 3 patients (7%) had an antenatal diagnosis of placenta previa or normal placentation in the setting of a history of cesarean delivery.

**Table 1:** Demographic data

Demographics	
Demographic	Median (IQR)
Maternal age (years)	30 (30–36.8)
BMI (kg/m^2^)	33.0 (26.0–35.5)
GA (weeks)	34.8 (32.9–35.6)
Gravidity	4 (3–6)
Parity	2.5 (1–4)
Prior cesarean	2 (1–3)
Race/ethnicity	N (%)
Hispanic	21 (50%)
Non-Hispanic white	17 (40.5%)
Black	1 (2.4%)
Other/unknown	3 (7.1%)
Type of PAS (antenatal diagnosis)	N (%)
Previa only	1 (2%)
Accreta	19 (46%)
Increta	8 (19%)
Percreta	12 (29%)
Normal	2 (4%)

Demographic data presented include maternal age (in years), body mass index (BMI), gestational age (GA), gravidity, parity, and number or prior cesarean deliveries of all patients included in this study. Median is shown with associated interquartile range (IQR). Race/ethnicity presented as a percentage of the total number of subjects in the study. Type of placenta accreta spectrum (PAS) made by antenatal diagnosis preoperatively is shown as a percentage of the total number of subjects in the study

The median gravidity and parity were four (IQR 3–6) and 2.5 (IQR 1–4) with a history of two prior cesarean sections (IQR 1–3). Eighty-three percent of all pregnancies (*n* = 35) were complicated by placenta previa. Median gestational age at delivery was 34.8 weeks (IQR 32.9–35.6).

A transverse skin incision was made in 43% (*n* = 18) and a vertical skin incision in 57% (*n* = 24) of cases. This was independent of placental location (*p* = 0.82), degree of placental invasion (*p* = 0.26), BMI (*p* = 0.37), number of prior cesarean sections (*p* = 0.65), and gestational age at delivery (*p* = 0.26). (Table 2). The decision to perform a transverse or vertical skin incision was based on provider preference. There were no preoperative criteria used to determine the type of skin incision used. All transverse incisions were Pfannenstiel incisions.

**Table 2:** Type of skin incision by preoperative patient characteristics

Preoperative factor	Transverse incision *N* = 18 (43%)	Vertical incision *N* = 24 (57%)	*P*-value
BMI (kg/m^2^)	30.3	30.8	0.37
Placental location			0.82
Previa	15	22	
Anterior or fundal	2	2	
Posterior	1	0	
Type of PAS (antenatal diagnosis)			0.26
Accreta	7	12	
Increta	6	10	
Percreta	2	9	
Previa only	1	0	
No PAS	2	0	
Median # prior CD	2.1	2.3	0.65
Median GA at delivery (weeks)	34.7	33.9	0.26

Transverse versus vertical skin incision as function of body mass index (BMI, measured in kilograms (kg) per meters (m) squared), placental location, type of placenta accreta spectrum (PAS) made by antenatal diagnosis preoperatively, median number of prior cesarean deliveries (CD), and median gestational age (GA) at delivery in weeks

Intraoperative data are presented in Table 3. There were significantly more ureteral stents placed in the vertical skin incision group than in the transverse skin incision group (*p* = 0.003), however there was no difference in placement of endovascular balloon catheters (*p* = 0.33). There was no significant difference in mean estimated blood loss (EBL) between the transverse (2.6 L) versus vertical (2.8 L) skin incision groups (*p* = 0.82). Transfusion requirements were not different between groups with average number of total units transfused at 5.06 in the transverse skin incision group versus 4.19 in the vertical skin incision group (*p* = 0.71).

**Table 3:** Intraoperative outcomes

Intraoperative outcome	Transverse incision *N* = 18 (43%)	Vertical incision *N* = 24 (57%)	*P*-value
Use of ureteral stent	2	14	0.003
Use of aortic balloon	15	22	0.33
Mean EBL (L) (SD)	2.6 (1.9)	2.8 (3.8)	0.82
Transfusion requirements			
Mean # pRBCs (SD)	3.28 (4.47)	2.79 (5.00)	0.75
Mean # platelets (SD)	0.28 (0.57)	0.25 (0.61)	0.88
Mean # FFP (SD)	1.33 (2.35)	1.00 (1.93)	0.62
Mean # cryoprecipitate (SD)	0.17 (0.38)	0.13 (0.35)	0.75
Mean total # products (SD)	5.06 (7.63)	4.19 (7.67)	0.71
Mean operative time (minutes) (SD)	180 (86)	238 (74)	0.03
Intraoperative complications			0.42
Cystotomy	5	3	0.22
Ureteral Injury	1	2	0.72

Intraoperative surgical outcome data presented include use of ureteral stents, aortic balloon, estimated blood loss (EBL) in liters (L) (with standard deviation (SD)), transfusion requirements with packed red blood cells (pRBCs), platelets, fresh frozen plasma (FFP), cryoprecipitate, and total number of products, operative time, and intraoperative complications including cystotomy and ureteral injury

The type of skin incision modulated a significant difference in mean operative time between the two groups with transverse skin incision requiring 180 min on average compared to vertical skin incision at 238 (*p* = 0.03). There was no difference in operative complications. The overall incidence of intraoperative unintended organ injury was 23.8% (*n* = 10). There were five cases of cystotomy in the transverse skin incision cohort versus three cases in the vertical skin incision cohort (relative risk (RR) 1.55; 95% CI 0.52–4.62, *p* = 0.42). There was one case of ureteral injury in the transverse skin incision cohort versus two cases in the vertical skin incision cohort (RR 0.67; 95% CI 0.06–6.69, *p* = 0.73). The relative risk of overall intraoperative organ injury was not statistically significant between the two groups (RR 1.55; 95% CI 0.52–4.62, *p* = 0.42).

Postoperative data are presented in Table 4. Postoperatively, there was no significant difference in the length of hospital stay between the transverse vs vertical skin incision groups (*p* = 0.64). There was no significant difference in average intensive care unit length of stay (*p* = 0.64) or postoperative opioid use (*p* = 0.22). From the time of delivery to the 6-week postpartum follow-up visit, there were no wound complications with the transverse skin incision cohort; in comparison, 2 cases of wound complications occurred in the vertical skin incision cohort, both of which were superficial wound separation (*p* = 0.16). There were no maternal or fetal deaths in either group.

**Table 4:** Postoperative outcomes

Postoperative outcome	Transverse incision *N* = 18 (43%)	Vertical incision *N* = 24 (57%)	*P*-value
Mean postop LOS (days) (SD)	4.8 (3.1)	4.4 (1.5)	0.64
Mean ICU LOS (days) (SD)	0.45 (0.69)	0.62 (1.07)	0.64
Mean postop opioid use (ME) (SD)	117 (96)	180 (131)	0.22
Wound complications	0	2	0.16

Postoperative surgical outcomes data presented included mean postoperative length of stay (LOS) in days, mean intensive care unit (ICU) LOS in days, mean postoperative opioid use measured by morphine equivalents (ME), and wound complications. There were no fetal or maternal deaths

## Discussion

We demonstrate no significant differences in objective measures of surgical morbidity, including EBL, blood transfusions, and unintended organ damage for planned cesarean hysterectomy for PAS as a function of skin incision, transverse versus vertical. Vertical skin incision was associated with longer operative time; however, there was no difference in length of hospital stay or post-operative morphine equivalents used. To our knowledge, there is no data evaluating the type of skin incision performed for planned cesarean hysterectomies. Transverse incision appears a reasonable option in appropriately selected patients when compared to a vertical skin incision.

Decreasing any possible risks associated with morbidity related to PAS must be assessed both independently and as part of multidisciplinary surgical checklists. With the increasing incidence of PAS, measures to optimize maternal and fetal outcomes at the time of surgery are critical.

Prior studies have addressed other ways to decrease maternal and neonatal morbidity, including the use of multidisciplinary teams in planning scheduled cesarean hysterectomies, preoperative placement of ureteral stents to minimize ureteral injury, and preoperative endovascular interventions such as balloon catheter or arterial embolization [16–21]. Two studies have independently concluded that when compared with standard obstetric care, the use of multidisciplinary teams consisting of maternal fetal medicine specialists, anesthesia, neonatologists, interventional radiologists, urologists, and nursing in preparing for scheduled cesarean hysterectomies significantly reduces rates of large-volume blood transfusions, reoperation rates within 7 days for complications, intensive care unit admissions, and prolonged postoperative hospital stays [16, 17]. A meta-analysis found similar results, however did not show any difference in postoperative hospital length of stay [18].

Studies evaluating the use of preoperative ureteral stents to reduce of risk of urologic injury at time of cesarean hysterectomy have yielded conflicting results [2, 19, 20]. A recent review of the evidence in 2019 by Collins et al. found that use of ureteral stents may be useful in reducing the risk of ureteral injury in cases of placenta percreta; however, the current evidence is not strong enough to recommend their routine use [2]. Crocetto et al. showed that ureteral stents in association with cesarean hysterectomy do not reduce the risk of urinary tract injury in cases of placenta accrete [19]. A systematic review in 2012 by Tam Tam et al. found that the use of ureteral stents significantly reduces the risk of urologic injury in cesarean hysterectomies [20].

Using endovascular interventions such as balloon catheters or arterial embolization for cesarean hysterectomies remains a controversial topic, as it is difficult to predict for which patients the benefits of these interventions outweigh the risks, and whether their use reduces transfusion requirements [2, 21]. A meta-analysis by D’Antonio et al. in 2019 found that while the use of endovascular interventions significantly reduced the risk of blood loss greater than 2.5 L, there was no difference in transfusion requirements [21]. This study found a complication rate of approximately 5%, however reliable data on complication rates for use of endovascular interventions at time of cesarean hysterectomy are lacking [21]. The recent review by Collins et al. concluded that large, prospective, controlled studies on the use of endovascular interventions are necessary to recommend their routine use at time of scheduled cesarean hysterectomy [2].

We demonstrate that the only statistically significant difference in surgical outcome was seen with mean operative time in that transverse skin incision was nearly 60 min less than for vertical skin incision. This finding may be explained by the higher frequency of ureteral stent placement in the vertical skin incision group, which may indirectly reflect a higher degree of difficulty of cesarean hysterectomies in the vertical skin incision group. Another contributing factor may be secondary to increased provider comfort with opening and closing transverse incisions than vertical incisions because the tissue is generally on less tension than vertical incisions, and the underlying bowel is better protected by the abdominal wall musculature during fascial closure with a Pfannenstiel incision than with a midline vertical incision. This outcome is important for numerous reasons. The first is that with the increasing incidence of pregnancies affected by PAS, resource utilization becomes integral. While the vertical skin incision group had more ureteral stents placed, there were no differences in ureteral injury between both groups. Shorter operative time signifies less cost to the patient and the hospital, more operating room availability for other uses, and greater availability of surgeons, anesthesiologists, and other personnel. Second, shorter operating times may yield greater patient safety and satisfaction due to less time under anesthesia and therefore fewer anesthesia-related risks [22].

Others have described higher opioid use in patients who underwent vertical skin incision and conclude that a vertical abdominal incision is more painful than transverse abdominal incisions [23]. Our sample size may not have allowed assessment of all outcome variables, such as a difference in postoperative wound separation which has been described with vertical skin incisions because of the notion that incisions on greater tension are at greater risk of wound separation [23, 24].

There are no studies that have addressed perioperative outcomes based on the type of skin incision at the time of scheduled cesarean hysterectomy, transverse versus vertical. The review by Collins et al. acknowledges that there is no evidence to guide recommendations for type of skin incision, and consensus opinion concludes that type of skin incision should be left to the operating team [2]. Our study is the first that is designed to specifically answer this question.

One of the strengths of this study is that the type of skin incision performed was independent of patient BMI, placental location, type of abnormal placentation, gestational age, and number of prior cesarean sections. Therefore, the number of potentially important confounders is limited, as some of these variables alone have been shown to affect intraoperative outcomes, including blood loss, injury to surrounding organs, and length of surgery [25–28].

We recognize that our study has limitations. First, its retrospective nature captures a heterogenous disease (PAS). Nearly half of the patients in this study had placenta accreta, which is associated with less severe surgical morbidity in comparison to deeper degrees of invasion as is seen with placenta increta or placenta percreta. Secondly, our analysis was specific to a single health care system at a large, academic, tertiary care hospital that serves a diverse population; however, this could potentially limit the applicability of our findings. Third, varying surgical skill among surgeons and degree of difficulty of each cesarean hysterectomy may account for differences in length of time of surgery. For those cases in which the surgeon perceived the preoperative degree of difficulty to be high may have been more likely to perform a vertical skin incision. This limitation may be supported by the fact that significantly more ureteral stents were placed in the vertical skin incision group. Fourth, the small sample size may not have enough statistical power to identify significant differences in outcome metrics or morbidity. We were unable to fully control for all relevant confounding variables. Lastly, we had limited ability to evaluate differences in outcome metrics or safety given our small sample size and unable to account for patient-driven and provider-driven decisions related to skin incision.

## Conclusion

Placenta accreta spectrum is a condition with increasing incidence and maternal morbidity. Pre-, intra- and post-operative techniques to attenuate risk are essential. Our study suggests that transverse skin incision in our cohort is associated with similar surgical outcomes and similar rates of maternal morbidity, while decreasing operative time. Larger studies are needed to elucidate surgical methods and measures to optimize standard of care for this high-risk patient cohort. This study postulates that the difference in operative time could be clinically relevant, and powering a future study to investigate this outcome will be our next step.

## Data Availability

The datasets during and/or analyzed during the current study available from the corresponding author on reasonable request.

## Abbreviations

BMI: Body mass index
CD: Cesarean delivery
EBL: Estimated blood loss
FFP: Fresh frozen plasma
GA: Gestational age
ICU: Intensive care unit
IQR: Interquartile range
IRB: Institutional review board
Kg: Kilogram
L: Liters
LOS: Length of stay
M: Meters
ME: Morphine equivalents
PAS: placenta accreta spectrum
pRBC: Packed red blood cells
RR: Relative risk
SD: Standard deviation
